# AIMAR survey on COPD phenotypes

**DOI:** 10.1186/2049-6958-9-16

**Published:** 2014-03-17

**Authors:** Maria Sandra Magnoni, Andrea Rizzi, Alberto Visconti, Claudio F Donner

**Affiliations:** 1 Medical Department, GlaxoSmithKline, Verona, Italy; 2 AIMAR (Interdisciplinary Association for Research in Lung Disease), Arona, NO, Italy; 3 Mondo Medico Multidisciplinary and Rehabilitation Outpatient Clinic, Borgomanero, NO, Italy

**Keywords:** COPD, Exacerbations, Online survey, Phenotypes

## Abstract

**Background:**

COPD is characterized by considerable diversity in terms of clinical signs and symptoms, physiopathological mechanisms, response to treatment and disease progression. For this reason, the identification of different patient subgroups (or possible phenotypes) is important both for prognosis and for therapeutic objectives. Based on the foregoing, AIMAR has decided to conduct a survey on the perception of the prevalence of the different clinical COPD phenotypes/subtypes in the clinical practice of physicians who treat patients with chronic obstructive pulmonary disease, and on their therapeutic objectives.

**Methods:**

The survey consisted of 19 multiple-choice questions, compiled through a form published online. All the data and answers entered into the system were checked for consistency and completeness directly online at the time they were entered, and each respondent could only complete the questionnaire once.

**Results:**

The survey took place from May through October 2012. A total of 1,434 questionnaires (60% of the sample approached) were eligible for analysis, broken down as follows: 537 pulmonologists, 666 general practitioners (GPs), 72 internal medicine specialists, 36 allergists, 30 geriatricians, 93 other specialists. The results show that a significant proportion of GPs (33%) identified more than 50 patients in their practices with a diagnosis of COPD. Although most patients are or have been in treatment with a long-acting bronchodilator, the most common reasons for seeing a GP or a specialist were exacerbations and worsening of the symptoms, suggesting the importance of an appropriate background therapy in order to reduce the risk of disease instability. The frequent exacerbator phenotype was the most commonly found phenotype in clinical practice (by 75% of specialists and 66% of GPs); patients with a prevalent phenotype of chronic bronchitis were reported more often by GPs, while specialists reported a higher number of patients with a prevalent phenotype of emphysema.

A medical history of exacerbations and the extent of deterioration of the spirometry parameters were considered to be the major indicators for COPD severity and clinical risk. In managing the frequent exacerbator phenotype, the therapeutic objectives – both for GPs and for specialists – included reducing airway inflammation, improving bronchial dilation, and reducing pulmonary hyperinflation. For this type of patients at high clinical risk, specialists selected a first-line therapeutic option based on a predetermined combination of an inhaled corticosteroid (ICS) and a long-acting β_2_-agonist bronchodilator (LABA) and a second-line three-drug therapy (combination of ICS and two long-acting bronchodilators), while GPs’ choices are more diversified, without a clear-cut prevalence of one type of treatment. In patients with COPD and concomitant cardiovascular diseases, frequently observed in clinical practice by all physicians, the combination of ICS and LABA was considered the first-choice option by the highest proportion of GPs (43%) and specialists (37%), while a smaller number of specialists (35%) opted for the long acting muscarinic antagonists (LAMA). Both GPs and specialists believe that therapeutic continuity is of primary importance for the achievement of clinical outcomes with all classes of drugs.

**Conclusions:**

A good knowledge of COPD has been observed in a high percentage of GPs, indicating an increased awareness of this disease in Primary Health Care. The frequent exacerbator phenotype is viewed by all physicians as the most prevalent in clinical practice, bearing a high risk of hospitalization. For specialists, therapeutic measures aimed at reducing the number and severity of exacerbations are primarily based on the combination of inhaled corticosteroid and bronchodilator, presumably because of the complementary pharmacological action of its components, whereas while GPs’ choices tend to be more diversified. Adherence to medication regimens is of the essence for the achievement of clinical outcomes.

## Background

Chronic obstructive pulmonary disease (COPD) is a complex, multi-component disease with a significant heterogeneity with respect to clinical presentation (symptoms, exercise tolerance, exacerbation susceptibility), radiological features, mechanisms of airflow limitation and disease progression, independently of the degree of airflow limitation [1].

Within the scientific community there has been a growing interest in categorizing this clinical and physiopathological heterogeneity into COPD “phenotypes”. The concept of phenotype is not new: in the mid-20^th^ century COPD was classified as “phenotype A”, characterized by findings of emphysema, and “phenotype B”, characterized by chronic bronchitis, on the basis of clinical, radiological and physiopathological evidence [2]. These two types were the extremes of a spectrum and often coexist to different extent in most patients. In recent years the advances of imaging diagnostics (high resolution computerized tomography, MRI) have considerably fostered a renewed interest in the classification of patients by clinical-pathological phenotypes, which is driven by the development of new studies to define the clinical phenotypes of COPD [3-7].

An essential contribution to knowledge in this field came from the study of the ECLIPSE [7] cohort, one of the largest and best characterized within the scope of COPD studies: 2,180 patients and 566 controls, smokers and non-smokers, were followed up for three years in order to define clinically relevant COPD subtypes and to identify parameters and bio-markers able to predict disease progression. One of the first analyses [8] of the longitudinal data gathered from the ECLIPSE cohort led to the identification of a distinct COPD phenotype at higher risk of exacerbation (“frequent exacerbator”), which is stable over time, similar across GOLD stages, characterized by inherent susceptibility to triggers like viral infections, clinically predictable and identifiable through the clinical history. In fact, a medical history of exacerbations was found to be the predictive factor most closely correlated with their frequency.

More recently, it has been observed that the patient subgroup accounting for approximately 16% of the cohort had constantly elevated levels of markers of systemic inflammation, correlated with an increased risk of death and exacerbation compared to patients with less inflammation. It has been suggested that the constant presence of systemic inflammation, flared up by repeated exacerbations [9], identifies a new, distinct phenotype of COPD patient, who might be treated with specific therapeutic strategies [10].

The heterogeneity of COPD may make this disease very complex to manage, as the different phenotypes could have diverging prognosis or treatment needs. Hence the interest in investigating the perception and impact of the different COPD phenotypes in the clinical practice of physicians who treat patients with chronic obstructive pulmonary disease. On this basis, AIMAR (Interdisciplinary Scientific Association for the Study of Respiratory Diseases) decided to launch a survey on this specific subject, with the help of an unconditional contribution from GlaxoSmithKline, in order to collect information on the following main aspects:

– Perception of the frequency of the different COPD phenotypes (frequent exacerbator, with prevalent emphysema, with prevalent chronic bronchitis, with marked reversibility).

– Management of the different types of COPD patients.

## Methods

On the basis of the objectives indicated above, the scientific committee that thought of the survey developed a questionnaire that acquired data through a form available online, which the respondents could complete at any time. The questionnaire included 19 multiple-choice questions and was addressed to the following specialists: pulmonologists, allergy specialists, geriatricians, internal medicine specialists and general practitioners. All the data and answers entered into the system were checked for consistency and completeness directly online at the time they were entered, and each respondent could only complete the questionnaire once. The data gathered in this way restored directly into a relational database (based on the proven open-sourceMySQL technology), and therefore were immediately available for queries even while the survey was still ongoing. The access to the data, mediated by a back office query and reporting system, allowed constant monitoring of the correct conduct of the survey; it was also possible to extract and check data on smaller data sets.

## Results

Of the 1,516 questionnaires collected (corresponding to 63,4% responders), 82 were incomplete or erroneously compiled, and therefore considered not valid, coming to a total of 1,434 questionnaires eligible for analysis (60% of the sample approached), broken down as follows: 537 pulmonologists, 666 general practitioners, 72 internal medicine specialists, 36 allergy specialists, 30 geriatricians, 93 other specialists (including anesthesiologists and resuscitators, cardiologists, ear, nose and throat specialists, allergy specialists, emergency physicians, physiatrists, oncologists, and thoracic surgeons).

### Epidemiology

**Figure 1:**
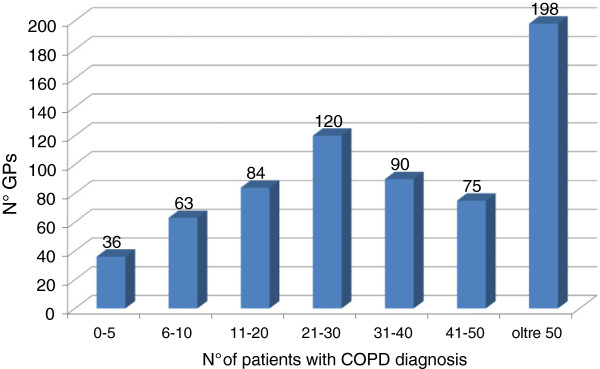
Number of patients with a COPD diagnosis out of those treated by GPs.

Against this backdrop, the results of the survey (Figure 1) indicate that approximately one third of GPs has identified more than 50 patients in their practices with a diagnosis of COPD, and 56% has identified more than 20 patients: this points to an increased awareness of this disease in Primary Health Care.

**Figure 2:**
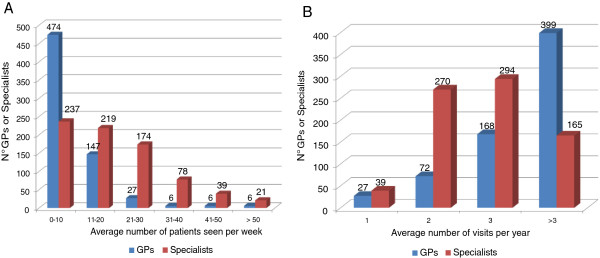
**Average number of patients per week (A) and average number of visits on the same patient per year (B).**

The average number of patients with COPD examined by the majority of GPs per week is lower than 10, in line with the stated number of patients, while specialists range between fewer than 10 (30%), 10 to 20 (28.5%) and more than 21 (22%) patients per week (Figure 2A). Out of 237 specialists who declared they see fewer than 10 patients per week with COPD, 40.5% (96) are pulmonologists; out of 219 specialists who said they see 10 to 20 patients, 72.6% (159) are pulmonologists; of those who said they see more than 21 patients, 52.5% (282) are pulmonologists.

As to the number of visits of individual patients per year, the results show a rather high frequency – at least three visits per year – for 85% of GPs (more than three visits per year in 60% of cases): the reasons of the physical examinations presumably range from disease monitoring to worsening of the symptoms or management of possible comorbidities. The majority of specialists see the same patient two or three times per year (Figure 2B).

Table 1 shows that the reason for not yet diagnosed patients to first see their GP is, in 48% of cases, symptoms of chronic bronchitis and in 15.7% of cases an exacerbation, while a not negligible proportion of patients (30%) sees their GP for other reasons *(among others: polyglobulia secondary to the respiratory disease, increased hematocrit, and symptoms of viscosity).* In the case of specialists, the main cause for first presenting for examination is not a worsening of the symptoms or diseases other than COPD, but other reasons (51%), probably in connection with hospitalizations due to respiratory failure [9].

**Table 1:** Most frequent cause for patients to first see their GP or a specialist

**Dyspnea & limitations**		**Exacerbations**		**Chronic bronchitis/cough**		**Disease other than COPD**		**Other**	
**GP (%)**	**Spec.(%)**	**GP (%)**	**Spec (%)**	**GP (%)**	**Spec. (%)**	**GP (%)**	**Spec. (%)**	**GP (%)**	**Spec. (%)**
0.45	5,8	15.7	19,5	48,2	21,8	4,9	1,5	30,6	51,1

**Figure 3:**
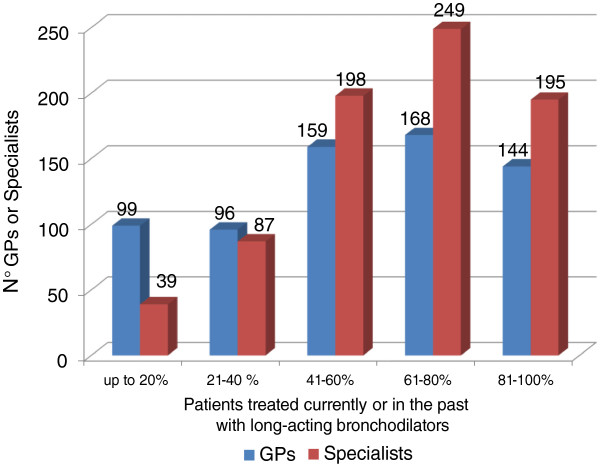
Patients with COPD treated currently or in the past with long-acting bronchodilators.

As shown in Figure 3, a vast majority of patients is or has been in treatment with a long-acting bronchodilator, which is the first step in the treatment process in the presence of persistent symptoms: in the event of unsatisfactory response to the therapy, worsening of the symptoms and/or exacerbations, the indications might exist for switching to a combination therapy by adding an ICS to the long-acting β_2-_agonist bronchodilator (LABA).

**Figure 4:**
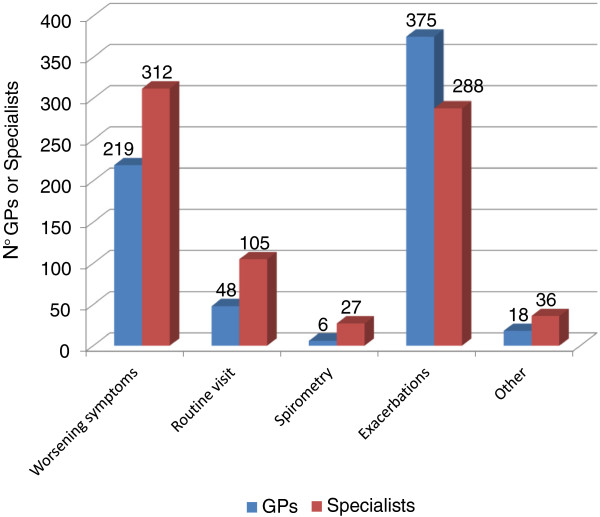
Causes for COPD patients to submit to subsequent examinations.

Within this context, the survey identified exacerbations and worsening of the symptoms as the most frequent causes of subsequent medical examinations, both for GPs (56% to 33% respectively) and for specialists (37% and 40% respectively) (Figure 4).

### Types of COPD patients or phenotypes

**Figure 5:**
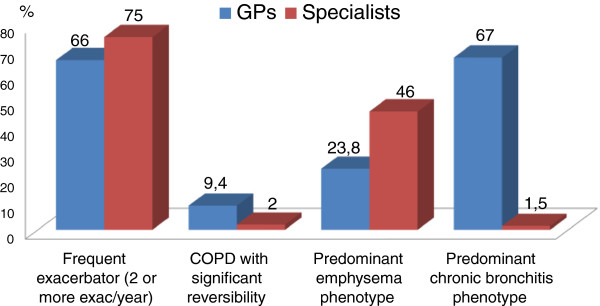
Types of patients most frequently seen by GPs and specialists in clinical practice.

As to the heterogeneity of COPD, virtually all physicians, whether GPs (84%) or specialists (92%), believe that there are different patient types or phenotypes. Figure 5 shows the types of COPD patients most frequently encountered in the clinical practice of the physicians participating in the survey: 66% of GPs report a high incidence of the frequent exacerbator phenotype (2 o more exacerbations per year, defined along the GOLD 2011 criteria) and of the phenotype characterized by prevalence of chronic bronchitis; on the other hand, among specialists the frequent exacerbator is found to be the most common phenotype by 75%, and the phenotype characterized by prevalence of emphysema by 46%. The type of patients with significant reversibility in the bronchodilation test is encountered rarely, not only by GPs, but also by specialists.

As to the reported prevalence of the different phenotypes in everyday clinical practice, Table 2 shows that the “frequent exacerbator” is perceived as having a rather high prevalence and accounts for over 30% of the population of COPD patients according to 31% of GPs and 45% to specialists, and for 10 to 30% according to 44% of GPs and 41% of specialists. The “chronic bronchitis” phenotype is also considered to have high prevalence, accounting for more than 30% of the COPD patient population seen in clinical practice according to more than half (53%) of both GPs and specialists. Conversely, the emphysematous is considered to have low prevalence by 60% of GPs (<10%), while according to more than half of the specialists (54%) its prevalence ranges between 10 and 30%. The majority of both GPs and specialists indicates the percentage of patients with significant reversibility as less than 10%.

**Table 2:** Prevalence of the different types of patients (phenotypes) in the COPD patient population treated by GPs and specialists

	**Frequent exacerbator phenotype**		**Predominant emphysema phenotype**		**Predominant chronic bronchitis phenotype**		**COPD with significant reversibility**	
**Prevalence**	**GP (%)**	**Spec. (%)**	**GP (%)**	**Spec. (%)**	**GP (%)**	**Spec. (%)**	**GP (%)**	**Spec.(%)**
<10%	23,8	13,2	60,3	31,6	13,5	13,2	65,3	69,5
10-30%	44,6	41,4	33,7	54,3	32,8	33,2	25,6	27
<30%	31,5	45,1	5,3	14	53,8	53,5	9	3,5

### Categorization of COPD patients: severity indicators, risk of hospitalization

**Figure 6:**
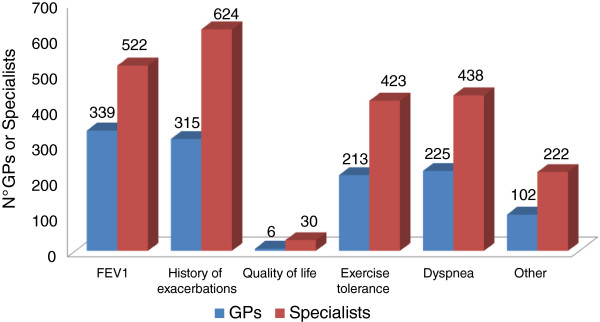
Indicators for COPD severity classification.

**Figure 7:**
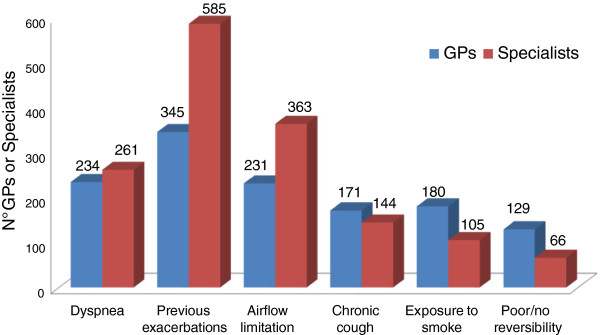
Top predictive factors of the risk of COPD exacerbation (max two options).

With regard to the key indicators for COPD patient severity classification, nearly all the specialists (81%) mentioned a medical history of exacerbations, followed by spirometry parameters (68%), dyspnea and low tolerance to effort; for GPs, spirometry parameters (51%) and a history of exacerbations (47%) are the top indicators (Figure 6). Most specialists – unlike GPs – indicate a clinical history of exacerbations as the predictive factor most closely correlated with their frequency (Figure 7).

As COPD exacerbations are crucial in this disorder (not only do they impact significantly the patient’s quality of life and evolution of the disease, but they can also be associated with cardiovascular complications and death [11-16], causing a substantial increase in health care costs [17]), we investigated how the different phenotypes are viewed in terms of risk of hospitalization. Table 3 shows that the frequent exacerbator phenotype is considered as the one at highest risk by most clinicians (76% of specialists and 53% of GPs), unlike the phenotype with significant reversibility in the bronchodilation test, concurrently considered at low risk. For the phenotype with prevalence of emphysema, as well as for that with prevalence of chronic bronchitis, the risk level is considered medium by the majority of physicians.

**Table 3:** Types of COPD patients considered at highest risk of hospitalization

	**Frequent exacerbator phenotype**		**Predominant emphysema phenotype**		**Predominant chronic bronchitis phenotype**		**COPD with significant reversibility**	
**Risk of hospital.**	**GP (%)**	**Spec. (%)**	**GP (%)**	**Spec. (%)**	**GP (%)**	**Spec.(%)**	**GP (%)**	**Spec. (%)**
Low	12,6	7,8	27,5	22,4	27,5	30	83	80,8
Medium	34,2	16	44,4	60,5	56	46,5	15	16,4
High	53,5	76	27,5	17	15,3	23,4	1,6	2,7

Another important aspect in assessing the COPD patients’ clinical picture is the presence of comorbidities, particularly of a cardiovascular nature, widely recognized both by GPs and by specialists (data not shown).

### The therapeutic approach: objectives and classes of drugs

**Figure 8:**
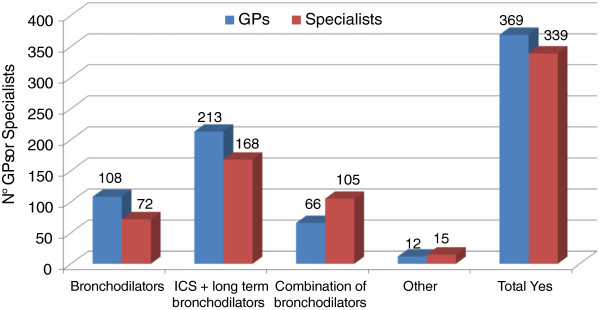
Indication of a pharmacological approach potentially effective for all COPD patients.

While recognizing the existence of different COPD patient types or phenotypes, approximately one half of the physicians (55% of GPs and 48% of specialists) believe that there is a standard pharmacological approach that is effective for all patients, i.e. the combination of inhaled corticosteroid (ICS) and bronchodilator. This is considered by both specialists and GPs the most effective treatment, followed by a combination of two bronchodilators according to specialists, and a single bronchodilator according to GPs (Figure 8).

**Figure 9:**
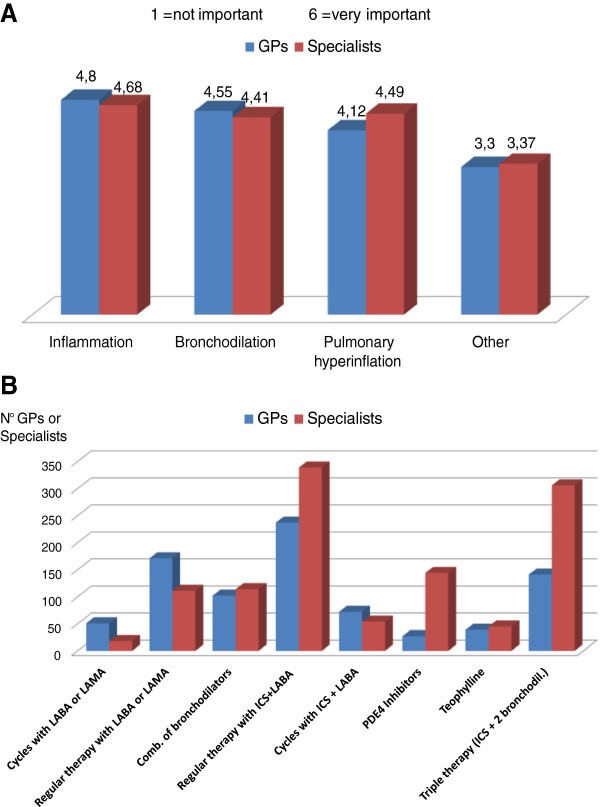
Role of physiopathological mechanisms (A) and therapeutic approach (B) in COPD patients with frequent exacerbations.

If we now focus on the treatment of frequent exacerbators, the physiopathological mechanisms considered by both GPs and specialists to be important in achieving the therapeutic effect are reduction of the airways inflammation, increased bronchodilation, and reduction of pulmonary hyperinflation, which is responsible for low tolerance to physical effort (Figure 9A). Consistently with the above, 44% of specialists indicate as first-choice therapy a fixed combination of ICS + LABA, which has a complementary action on the different physiopathological components (inflammation and bronchial obstruction), and 40% of them favor the three-drug therapy consisting of a combination of two bronchodilators plus ICS (Figure 9B). Therefore, for this type of patients at high clinical risk of exacerbations most specialists recommend the additional benefit of an ICS as opposed to bronchodilator therapy alone, in line with the evidence reported in literature [18-24] and incorporated in the guidelines [1,25].

The therapeutic choices of GPs are more diversified, although a higher proportion favors an ICS containing regimen (35% opt for the ICS + LABA combination, 21% for three-drug therapy), compared to individual bronchodilators (25% LABA or LAMA, 15% a combination of two bronchodilators). It should be noted, however, that one quarter of GPs opt for bronchodilator monotherapy even in patients at high risk of exacerbation (Figure 9B).

**Figure 10:**
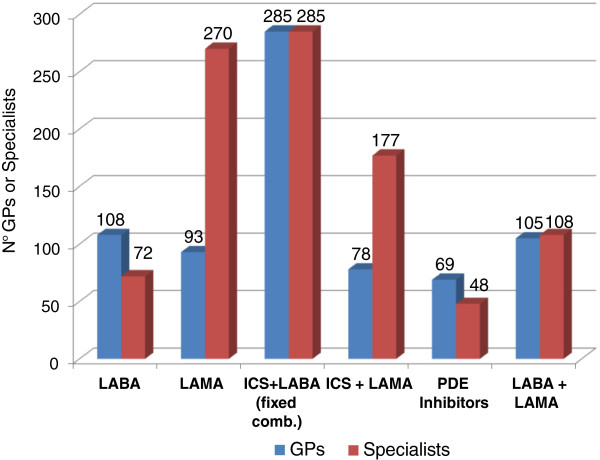
Therapeutic choices in patients with COPD and cardiovascular comorbidity.

As stated above, COPD patients tend to have a fairly high level of comorbidity: heart failure, coronary artery disease, diabetes, bone metabolism alterations, and depression; weight loss, nutritional disorders, and skeletal muscle dysfunctions are also well recognized extra-pulmonary effects of COPD [26-28]. This means that a multitasking treatment may be required, i.e. a treatment aimed at different objectives at the same time. In this regard, an important aspect is drug tolerability, and particularly cardiovascular tolerability. Figure 10 shows that for this type of patients the largest proportion of GPs and specialists (43% and 37% respectively) opt for the fixed ICS + LABA combination, and a significant proportion of specialists (35%) favours LAMAs.

**Figure 11:**
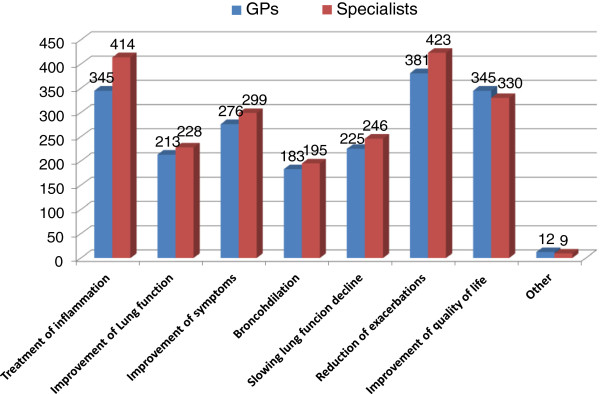
Therapeutic objectives in patients with asthma and a scarcely reversible obstruction (max three options).

As well known, a proportion of asthmatic patients may evolve into clinical pictures related to COPD [29-31], particularly if they are exposed to smoke or occupational dusts. Inadequate lung development at birth may also make the respiratory tree more vulnerable to external agents (e.g. smoke, viral infections), and be a risk factor for early development of a scarcely reversible obstruction process as well as of mixed or overlapping forms of asthma and COPD. For these forms of overlap, treatment of the underlying inflammation and reduction of exacerbations were considered by both categories of physicians as the primary objectives of therapy, along with application of symptoms and general improvement of patients’ quality of life (Figure 11).

### Therapeutic continuity

**Figure 12:**
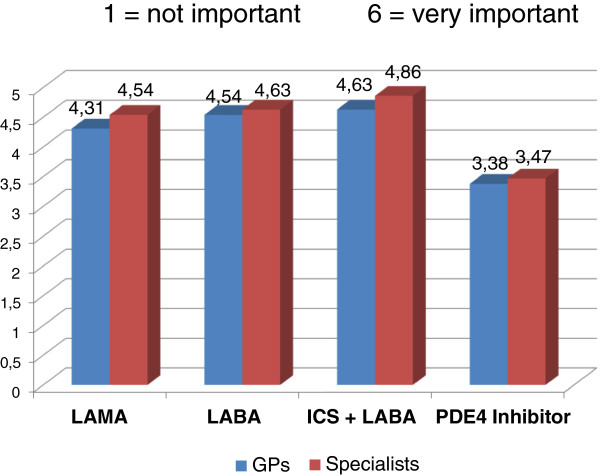
Importance of therapeutic continuity.

Within the context of chronic disease like COPD, both GPs and specialists consider therapeutic continuity to be essential for all classes of drugs in order to achieve the clinical outcomes (Figure 12).

## Discussion

COPD prevalence data, in relation to a known chronic bronchitis or emphysema diagnosis, indicate that this disorder affects around 4-6% of the general population [1], corresponding to approximately 3 million patients in Italy. This figure almost certainly underestimates the real size of the problem: in Italy, like in the rest of the world, COPD is widely under-diagnosed, often goes untreated until the advanced stages, and appears to be a major issue even among young adult [32,33].

Against this backdrop, the results of the survey indicate that a high percentage of GPs (approximately one third of them identified more than 50 of their patients with COPD), indicating an increased awareness of this disease in Primary Health Care.

Hopefully, this will result in greater attention to alarming symptoms and greater use of diagnostic tools, which would allow patients to be treated early on and to slow down their evolution towards disability. On the other hand, the fact that a significant percentage of GPs (44%) diagnosed with COPD fewer than 20 patients (much below all epidemiological data) suggests a need to continue to offer educational programs in this field. As it is well known, COPD can go undetected for a long time, because patients initially tend to minimize or ignore symptoms like cough and increased phlegm, which they may consider as a “normal” consequence of smoking, and get used to the difficulties caused by the disease by reducing their exercise. This is why they often first present for examination in an advanced stage of the disease [34]. These considerations emphasize the strategic role of GPs in proactive case finding, using, among other means, specific questionnaires administered to patients at risk, such as smokers.

While a large majority of patients is or has been in treatment with a long-acting bronchodilator, the most common causes of the medical examination are exacerbations and worsening of the symptoms, both for GPs and for specialists: this confirms the importance of an appropriate background therapy in order to reduce the risk of disease instability and use of healthcare resources alongside with compliance to inhalation therapy. To note, in patients with chronic bronchial obstructive disease compliance with inhalation therapy is often low, and errors in the use of inhalators are very common [35-40]: in this regard, physicians can play an essential role in patient education on therapeutic adherence, including the correct use of devices, thus improving disease management [41].

Virtually all physicians, both GPs and specialists, believe that there are different types or phenotypes of patients. The “frequent exacerbator” phenotype is one of the most commonly seen both by GPs and specialists in everyday clinical practice, with an estimated prevalence of more than 30% of the COPD patient population according to over one third of the physicians. The patient phenotype with prevalence of chronic bronchitis is more commonly reported by GPs (estimated to be more than 30% by approximately half of the physicians), while the phenotype with prevalence of emphysema is more commonly reported by specialists. The fact that the type of patients with significant reversibility to the bronchodilation test is encountered rarely presumably reflects the infrequent use of spirometry. It should be specified that data recently emerged from the ECLIPSE study have demonstrated that in many COPD patients reversibility is not a stable characteristic, and therefore does not define a phenotype [42].

With regard to COPD staging, a medical history of exacerbations and the extent of deterioration of spirometry parameters are considered to be top indicators for COPD severity. A primary role is attributed to disease exacerbations particularly by specialists, in line with scientific evidence which has conclusively demonstrated that spirometry parameters alone (FEV_1_) do not reflect the complexity of the disease and are insufficient to classify its severity [1]. In fact, the guidelines and recommendation documents advocate a multidimensional patient assessment, in which the measurement of bronchial obstruction is complemented with an evaluation of the risk of exacerbations and the presence of comorbidities [1,25]. To note, the 2013 update of GOLD guide-lines indicate that even one hospitalization for COPD exacerbation should be considered high risk [1].

The frequent exacerbator phenotype is considered at highest risk of hospitalization by both categories of physicians. Furthermore, knowing the frequency of exacerbations is viewed as essential to determine the clinical risk: in this respect, it is interesting to note that most specialists – unlike GPs – indicate a clinical history of exacerbations as the predictive factor most closely correlated with their frequency, in line with the findings of the ECLIPSE study [8] and with evidence from everyday clinical practice. It is worth mentioning that a recent study has demonstrated prospectively that COPD patients are able to estimate accurately the frequency of the exacerbations they experienced during the previous year [43]. The reliability and “strength” of their recollection allow the different types of patients – frequent exacerbators and non-frequent exacerbators - to be identified on the basis of medical history.

Another important aspect in assessing COPD patients’ clinical picture is the presence of comorbidities, particularly of a cardiovascular nature, widely recognized both by GPs and by specialists. The preliminary data of a cross-sectional European study have demonstrated that the prevalence of reduced air flow in a sample of 1,803 patients with cardiovascular disease treated on an outpatient basis is 30.6%; interestingly, only 29.4% of these had been previously diagnosed with COPD [44]. Therefore, a reduced air flow compatible with COPD is commonly found and under-diagnosed in patients with cardiovascular conditions. The presence of comorbidities or complications is relevant in determining COPD evolution and prognosis: it is estimated that approximately 50% of COPD patients die of cardiovascular causes, and the risk of death and hospitalization is higher the more severe the airway obstruction, regardless of whether the patient is a smoker or a non-smoker [45,46].

While recognizing the existence of different types or phenotypes of COPD patients, approximately half of the physicians believe that there is a standard pharmacological approach, which is effective for all patients. This approach is a combination of inhaled corticosteroid and bronchodilator, presumably because of the complementary pharmacological action of its components.

Taking therapeutic measures aimed at reducing the number and severity of exacerbations clearly results in savings in financial, social and health care resources [17]. In the treatment of frequent exacerbators, both GPs and specialists indicate reduction of airway inflammation, bronchodilation and reduction of pulmonary hyperinflation, which is responsible for low tolerance to physical effort, as essential to achieve the therapeutic effect. Accordingly, for this type of patients at high clinical risk, specialists sculpt primarily for regimens containing inhaled corticosteroid in fixed combination with LABA (or a three-drug therapy in combination with two bronchodilators), in line with evidence reported by literature and incorporated into the guidelines, while GPs’ choices tend to be more diversified, without a clear-cut prevalence of one type of treatment.

In patients with COPD and other concomitant disorders, frequently of a cardiovascular nature, special attention should be given to treatment tolerability: a fixed ICS + LABA combination is the first-line option according to the highest proportion of GPs and specialists, while a fairly large number of specialists opt for LAMAs. It is worth recalling that long-term clinical studies conducted on large populations of patients, many of whom had cardiac disorders and were in treatment with anti-arrhythmic agents, demonstrated a good tolerability profile of the ICS + LABA combination, at cardiovascular [47,48] as well as bone tissue level [49]; in general, the same can be said of long-acting bronchodilators [48,50].

In the forms of asthma evolving towards permanent obstruction, typical of patients who persist in the habit of smoking, or in mixed or overlapping forms of asthma and COPD, treatment of the underlying inflammation and reduction of exacerbations are considered by both categories of physicians as primary objectives of the therapy. Interestingly, the PLATINO study, a multicenter population-based survey carried out in five Latin American cities, report that the COPD-Asthma overlap is associated with increased severity, as indicated by the higher risks for exacerbations and hospitalizations compared to those with COPD [33].

Both GPs and specialists believe that therapeutic continuity is of the essence for the achievement of clinical outcomes with all classes of drugs. The literature reports examples of clinical worsening and failure to achieve therapeutic objectives due to low compliance with continuous treatment. A *post hoc* analysis of the TORCH study demonstrated that compliance is associated with a significant reduction of the risk of death and hospitalization due to exacerbations in COPD patients, regardless of treatment [51]. Another example is the observation that discontinuation of inhaled corticosteroid in patients with moderate-to-severe COPD stabilized with fluticasone/salmeterol combination led to a fast and significant deterioration of the function and symptoms and to increased exacerbations, confirming the role played by this class of drugs in the background treatment of COPD [23,24].

## Conclusions

In conclusion, a good knowledge and awareness of COPD has been observed in a high percentage of GPs. The frequent exacerbator phenotype is viewed by both specialists and GPs as the most prevalent in clinical practice, bearing a high risk of hospitalization. For specialists, therapeutic measures aimed at reducing the number and severity of exacerbations are primarily based on the combination of inhaled corticosteroid and bronchodilator, presumably because of the complementary pharmacological action of its components, whereas while GPs’ choices tend to be more diversified. Adherence to medication regimens is of the essence for the achievement of clinical outcomes.

## Competing interests

AV and CFD declare they have no competing interest to declare. MSM and AR report working as employees at Medical Department, GlaxoSmithKline.
